# Relationship between honesty-credit, specialty identity, career identity, and willingness to fulfill the contract among rural-oriented tuition-waived medical students of China: a cross-sectional study

**DOI:** 10.3389/fpubh.2023.1089625

**Published:** 2023-07-17

**Authors:** Xuewen Zhang, Bing Sun, Zhuang Tian, Bin Yu, Chao Wei, Ying Zhang, Canlei Zheng, Xuejun Chen, Qing Liu

**Affiliations:** ^1^School of Integrated Traditional Chinese and Western Medicine, Jining Medical University, Jining, China; ^2^School of Public Health, Jining Medical University, Jining, China

**Keywords:** career identity, honesty-credit, willingness to fulfill contract, RTMSs, specialty identity

## Abstract

**Background:**

The fulfillment of contractual obligations by rural-oriented tuition-waived medical students (RTMSs) to work in rural medical institutions after graduation directly impacts the improvement of rural health quality. This study aimed to not only quantitatively measure the direct impact of honesty-credit, specialty identity, and career identity on willingness to fulfill the contract of RTMSs but also to quantify the intermediary role of specialty identity and career identity between honesty-credit and willingness to fulfill the contract. The research results provided recommendations for the rural-oriented tuition-waived medical education (RTME) program to achieve its goal of training rural primary healthcare personnel.

**Methods:**

From March to May 2022, 1162 RTMSs were selected as the research objects. The honesty-credit, specialty identity, career identity, and willingness to fulfill the contract were quantitated using a self-completed questionnaire. Pearson's correlation analysis and structural equation modeling were used for statistical analysis and mediating effect evaluation.

**Results:**

A total of 455 (42.3%) RTMSs had high willingness to fulfill the contract, and honesty-credit had a significant direct positive effect on willingness (β = 0.198, *P* < 0.001), specialty identity (β = 0.653, *P* < 0.001), and career identity (β = 0.180, *P* < 0.001). In the intermediary path between honesty-credit and willingness, career identity [95% confidence interval (CI): 0.007–0.051] had significant mediating effects. Career identity (95% CI: 0.030–0.149) also had significant mediating effects between specialty identity and willingness, and specialty identity (95% CI: 0.465–0.760) had significant mediating effects between honesty-credit and career identity. These results strongly confirmed that honesty-credit, specialty identity, and career identity are early and powerful predictors of the willingness to fulfill the contract of RTMSs.

**Conclusion:**

The honesty-credit of RTMSs can predict their willingness to fulfill the contract early, significantly and positively. For the students who fail to pass the credit assessment for many times and have a strong tendency to default, their training qualifications should be canceled in time, so that students who are truly willing to serve rural areas can enter the project, and finally achieve the policy goal of “strengthening the rural primary medical and health system”.

## Introduction

The environment and facilities of the rural basic medical institutions have been significantly improved in China in recent years. However, the construction of human medical resources is relatively lagging behind, the quantity is insufficient, and the quality is not high, and therefore the team is unstable (1–3). As in many other countries (4, 5), the imbalance and unfairness in the allocation of doctors between urban and rural areas have plagued China. In 2005, the density of doctors in urban areas was more than twice as high as in rural areas (2.1 per thousand vs. 1.0 per thousand) (6). In 2011, the density gap between urban and rural doctors increased rather than decreased. The density of doctors in urban areas was 3.00/1000 and in rural areas 1.33/1000 (7). To address this issue, the National Development and Reform Commission of China took charge of launching and implementing the rural-oriented tuition-waived medical education (RTME) program in March 2010. The program aimed to train 300,000 general practitioners for rural communities within 10 years. Its primary focus was on the training of RTMSs who would serve in rural areas and provide safe, effective, convenient, and affordable basic medical services to rural residents (8, 9). As a result, government-funded tuition fees, accommodation fees, and living expenses were established for RTMSs. These students enter into a targeted employment agreement with the university and the local county-level health administration. The agreement entails a commitment to work in rural medical institutions for at least 3 years, following 5 years of undergraduate education and 3 years of standardized training.

Although the RTME program was brilliantly designed, the policy implementation was unsatisfactory. First of all, some students were not interested in the medical profession and applied for RTMSs out of “free” motivation or suggestions and requests from their families. They are pessimistic and disappointed about their future. Second, the preferential policy of directed employment after graduation from the RTME program led to a belief among certain students that employment was guaranteed as long as they gained admission to the university. Their negative and lazy mood of “learning or not learning, learning well or bad is the same (10).” directly affected the quality of graduates, and even went against the original intention of the government to implement the RTME program.

From 2010 to 2021, more than 70 medical universities in China enrolled more than 57,000 RTMSs (11). Can these RTMSs really take root in the countryside and provide quality health services to residents, as society hopes? How to ensure RTMSs work in designated rural medical institutions after graduation according to the agreement? All of them are unavoidable practical problems, which urgently need to be studied. In other words, the willingness of students to fulfill the contract becomes an essential factor affecting whether the RTME program can achieve the expected results.

Among 253 RTMSs trained by Chengdu Medical College in Sichuan Province in West China, 111 (45.5%) were not willing to fulfill contract to return to the countryside in future (8). Similarly, among 1673 RTMSs from three universities in Yunnan Province, 20.63% expressed no intention of practicing in rural communities. As they progressed in their education and gained more exposure to the rural medical environment, their likelihood of staying in the communities they had initially committed to practicing medicine decreased (12). This environment included factors such as less optimistic industry development, a lack of professional identity of rural doctors, and other negative factors, affecting their intention to perform their duties (11). Even though RTMSs passively complied with the contract to return to rural service for 3 years after graduation, their willingness to stay after the term of service was not high. Among 378 RTMSs in Chongqing, only 15.56% were willing to continue working at the grassroots level after the expiration of the agreement (13). Of 232 RTMSs in Shaanxi Province, up to 92.6% indicated that they would leave the countryside after the 6-year contract (14), which was higher than the turnover intention rate of 86.4% of local rural doctors (unfunded training) (15).

The factors influencing the intention of RTMSs in rural areas to provide basic medical services in future included various aspects, including the negative influence of social status and industry development, leading to a low sense of their specialty and career identity. The theory of vocational education holds that honesty-credit education serves as the fundamental element in all forms of education. It plays a crucial role in assisting students in establishing and developing specialty identity, subsequently leading to the development of their career identity and motivation (16). However, the RTME program lacks strong measures to address the breach of contract behaviors of RTMSs, resulting in students with weak honesty-credit awareness exhibiting a clear deficiency in their specialty identity and career identity (12–17). According to the occupational commitment theory, enhancing students' level of specialty identity and their satisfaction in their chosen field can effectively guide them in establishing a strong connection between themselves and their major. This, in turn, facilitates their active engagement in specialty learning behavior, ultimately improving the quality of talent training and enhancing their vocational competitiveness and career identity (18). Applying the theory to the willingness to fulfill the contract of RTMSs and analyzing the reasons, it was found that most RTMSs in Zhejiang Province did not choose medical majors and universities according to their own preferences. Consequently, this mismatch resulted in a notable absence of learning efficiency and a lack of career motivation among these individuals (17). At the same time, the social identity of rural doctors was not clear, and rural hospitals were far lower than urban hospitals in terms of salary, management system, career prospects, workload, promotion, continuing education opportunities, and career confidence, which also led to the lack of career norms and career identity, prompting rural doctors to move to private hospitals or engage in other industries after the term of service (12–17). In addition, studies have found that some RTMSs were prone to depression and anxiety due to dissatisfaction with their professional identity and directional employment in rural areas, which affected the smooth progress of their medical study (19).

During the epidemic, rural doctors faced the daunting task of carrying out complex and demanding work such as active surveillance, screening and follow-up, despite the scarcity of medical facilities, medicines, and training opportunities to effectively respond to public health emergencies. Accordingly, first-line village doctors, who were generally older, faced a higher risk of occupational exposure and infection compared with the general population. Shockingly, 95.5% of these doctors suffered from insomnia, anxiety, and fear, significantly impeding their ability to continue their work and affecting the stability and sustainability of rural healthcare (20).

**Figure 1:**
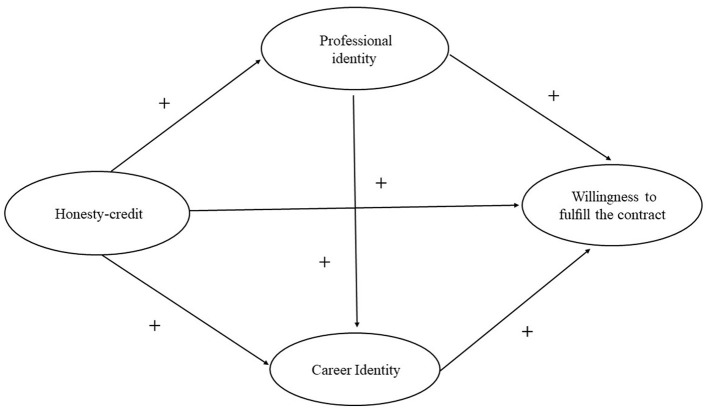
Theoretical model and hypotheses.

To sum up, the fulfillment of contracts by RTMSs as scheduled after graduation is key to the long-term sustainable development of the RTME program. It is directly related to the improvement of rural healthcare quality and the maintenance of the health of rural residents. By understanding the willingness of RTMSs to serve the grassroots level and its influencing factors, the expected objectives of the project can be better achieved. At present, the academic research on RTMSs mainly focuses on the theoretical exploration and evaluation of training programs, curriculum systems, post-graduation salary, working environment, and promotion space. However, the research on the willingness to fulfill the contract of RTMSs is insufficient and needs further study. At the same time, the existing studies mostly used Chi-square test, *T*-test, ANOVA, multiple linear or multiple logistic regression analysis, and other methods to analyze the willingness to fulfill the contract of RTMSs and its influencing factors (18, 21–30). Compared with the aforementioned studies, structural equation models (SEMs) can not only measure the correlation between study variables but also mine the correlation between potential variables, and even explain the causal relationship between variables. Therefore, this study not only quantitatively explored the direct impact of specialty identity, career identity, and honesty-credit on RTMSs' willingness to fulfill the contract but also used SEM to test the mediating role of specialty identity and career identity between honesty-credit and willingness to fulfill the contract. Furthermore, a double-intermediary data model was conducted, as presented in Table 1 and Figure 1, to illustrate these relationships. The research results provided suggestions and opinions for continuously improving the specialty identity, career identity, and professional interest of RTMSs. These recommendations include fostering correct professional beliefs, cultivating a sense of integrity, and encouraging long-term commitment to grassroots medical and health services. By implementing these measures, the RTME program can effectively achieve its talent training goal of “going down, using up, staying, and doing well,” ensuring sustainable and successful outcomes.

**Table 1:** Theoretical hypotheses.

**Hypotheses**
1. RTMSs' ***honesty-credit*** positively impacts ***willingness to fulfill the contract***.
2. RTMSs' ***specialty identity*** positively impacts ***willingness to fulfill the contract***.
3. RTMSs' ***career identity*** positively impacts ***willingness to fulfill the contract***.
4. RTMSs' ***honesty-credit*** indirectly positively impacts ***willingness to fulfill the contract*** through the mediating effect of ***specialty identity***.
5. RTMSs' ***honesty-credit*** indirectly positively impacts ***willingness to fulfill the contract*** through the mediating effect of ***career identity***.
6. RTMSs' ***honesty-credit*** indirectly positively impacts ***career identity*** through the mediating effect of ***specialty identity***.
7. RTMSs' ***specialty identity*** indirectly positively impacts ***willingness to fulfill the contract*** through the mediating effect of ***career identity***.

## Methodology

### Setting and participants

In East China, Shandong Province implemented the RTME program in 2017 and welcomed its first targeted medical graduates in 2022. According to the method of stratified cluster random sampling, three medical universities were randomly selected as sample points (Jining Medical University, Binzhou Medical University, and Shandong First Medical University) from the four designated medical universities chosen to train RTMSs in Shandong Province. Then, these universities were stratified according to senior (nine classes), middle (nine classes), and junior grades (nine classes), and 1200 people were randomly selected and included in the survey of this study. Thirty-eight students were excluded based on the following exclusion criteria: (1) students who refused to participate; (2) students who failed to register at the beginning of the freshman year; and (3) students who refused to sign the directed employment agreement. A total of 1,162 students were included to fill out the questionnaire, and 1,103 questionnaires were collected, resulting in a recovery rate of 94.92%. Among which 1076 questionnaires were deemed valid, yielding an effective recovery rate of 97.55%.

Ethical review was sought from the ethics committee of Jining Medical University, and the expert group issued ethical consent after reviewing the materials. Medical interventions were not involved in this study, and the data obtained from the findings could not be linked to individuals. Therefore, participants were informed about the anonymous nature of the study, and the purpose of the research before data collection commenced. Based on the verbal consent of the participants, data collection was carried out.

Since university students with high reading comprehension ability were the objects of the survey, the questionnaire information, including cover letters, general sociological characteristics, and scales, was collected using collective distribution on-site and self-filling questionnaires. At the same time, in the three universities, special personnel were arranged to be responsible for the issuance, recycling, and quality verification of questionnaires to ensure quantity and quality.

### Measures

Referring to the survey of career obstacles of Chinese university students (21), a questionnaire was designed, which consisted of five parts. The first part comprised the survey of demographic characteristics (age, sex, grade, and place of origin) and academic characteristics (learning cadres, learning objectives, learning motivation, and place of signing service). The second to the fifth parts were the scales of medical specialty identity, doctor career identity, and honesty-credit and willingness to fulfill the contract.

#### Honesty-credit

The honesty-credit education of RTMSs was not only the core of cultivating high-quality rural medical talents but also the key to constructing a harmonious doctor–patient relationship. The honesty-credit scale used in this study was adapted from Wu et al. (22) university students' honesty-credit-level questionnaire. We modified the scale to align with the characteristics of our research object and the purpose of the study. With Cronbach's α of 0.808 (22), the questionnaire included 4 dimensions, namely, medical students' own honesty-credit (1–6 questions), attitude to honesty-credit (7–13 questions), factors influencing university students' honesty-credit (14–19 questions), and methods to improve honesty-credit (20–25 questions). The questionnaire adopted a 5-point positive Likert score, and the higher the score, the stronger the honesty-credit.

#### Specialty identity

Specialty identity affected the learning psychology and professional ability of university students. Whether college students like and identified with their specialty became an important factor affecting their academic performance. The college student specialty identity scale prepared by Qin et al. (23) was used in this study to measure the specialty identity of RTMSs. The Cronbach's α was 0.729, which divided college students' specialty identity into four dimensions: cognitive (five items), emotional (eight items), behavioral (six items), and appropriateness (four items). Five points were scored using the Likert scale, the higher the score, the higher the specialty identity.

#### Career identity

The career identity scale for college students was compiled by Zhang et al. (24), and Cronbach's α of the scale was 0.866 (24). The scale included three dimensions: career benefit (six items), external influence factors (four items), and career motivation (six items). The scale was formed using the Likert scale, which ranged from 1 (completely disagreeing) to 5 (very agreeing). The higher the score, the stronger the sense of career identity.

#### Willingness to fulfill the contract

The willingness to fulfill the contract scale of RTMSs used in this study was adapted from the Chinese medical staff turnover intention scale and verified by preliminary investigation, and the Cronbach's α was 0.699 (25). The four items in the questionnaire were: “I want to leave the specialty of rural-oriented tuition-waived medicine and switch to other medical specialties”, “I often want to give up studying medical specialty”, “In the future, I will leave the orientated rural hospital during the term of my contract,” and “In the future, I will leave the medical industry during the term of my contract.” Using the Likert scale, the score ranged from 1 (very agree) to 6 (very disagree), with a total of 6 points. The higher the score, the more significant the willingness to fulfill the contract.

### Statistical analysis

First, the reliability and validity of the whole questionnaire were evaluated scientifically and accurately using the exploratory factor analysis (EFA) before the statistical analysis of variables. Second, the descriptive statistical analysis method was used to describe and analyze the demographic and academic characteristics of 1076 RTMSs, and the results were presented in terms of composition ratio. Third, the honesty-credit, specialty identity, career identity, and willingness to fulfill the contract of RTMSs were quantitatively analyzed, and the results were expressed as mean and standard deviation (SD). Then, pair-to-pair correlations among major variables were measured through Pearson's correlation and quantified into correlation coefficients. On this basis, the structural equation model (SEM) was used to further explore the relationships among the four dimensions of honesty-credit, specialty identity, career identity, and willingness to fulfill the contract. The maximum likelihood model based on bootstrap was applied to measure several key indicators of the model and data-fitting degree, including Normed Fit Index (NFI), goodness-of-fit index (GFI), comparative fit index (CFI), adjusted goodness-of-fit index (AGFI), incremental fit index (IFI), and Tucker–Lewis index (TLI). All these indexes were >0.90, indicating a favorable level of fit. The approximate root mean square error of approximation (RMSEA) of 0.067 was lower than 0.8, which proved to be in line with the data and assumptions, and it was an acceptable model.

### Reliability and validity

The EFA results showed that the Kaiser–Meyer–Olkin measure of the questionnaire was 0.960, >0.70, indicating a good possibility of data factor analysis. The Bartlett sphericity test showed a significant difference (χ^2^ = 45610.872, *P* < 0.001). The orthogonal rotation analysis of each factor load was carried out using the maximum variation coefficient method, and the load matrix results were obtained. The eigenvalues of the four evaluation indexes were all >1, and the cumulative variance contribution rate reached 65.285%, indicating that the validity of the questionnaire was not perfect but acceptable (26). Cronbach's α values of honesty-credit, specialty identity, career identity, and willingness to fulfill the contract were as high as 0.781, 0.828, 0.837, and 0.821, respectively, indicating good reliability (27).

## Results

### General demographic and academic characteristics

Table 2 presents the general demographic and academic characteristics of 1076 RTMSs. The investigated RTMSs ranged in age from 17 to 34 years, with an average age of (20.07 ± 1.678) years. The proportion of students from rural areas (59.3%) was more than that from urban areas (40.7%), and 39.0% were the only children of their parents. Among them, 15.2% of the respondents reached the admission score of clinical medicine undergraduates (non-public funded, non-rural directed employment), which was much higher than the admission score of RTMSs. Furthermore, 44.6% of these chose RTMSs because they loved medicine, 86% wanted to take the postgraduate examination after graduation, and 55.5% believed that the salary was the main reason that affected them to perform their duties in rural primary medical institutions in future.

**Table 2:** General characteristics of the respondents (*N* = 1076).

**General characteristics**	***N***	**%**
**Sex**		
Male	579	53.8
Female	497	46.2
**Grade**		
Senior	359	33.4
Middle	402	37.4
Junior	315	29.2
**Student origin**		
Rural	638	59.3
Urban	438	40.7
**Only child of parents**		
Yes	420	39.0
No	656	61.0
**Score of the college**		
Yes	164	15.2
No	912	84.8
**Reasons for applying for RTMSs**		
Love medicine	480	44.6
No employment pressure	227	21.1
Involuntary choice	142	13.2
Reduce the burden	94	8.7
Optional	70	6.5
Primary medical services	63	5.9
**Postgraduate entrance examination intention**		
Yes	925	86.0
No	151	14.0
**Factors affecting performance**		
Salary	597	55.5
Work content	229	21.3
Working environment	113	10.5
Promotion space	111	10.3
Other factors	26	2.4

### Descriptive analysis of study variables

The total score of honesty-credit, specialty identity, career identity, and willingness to fulfill the contract was 94.04 ± 10.25, 88.35 ± 14.26, 57.49 ± 9.79, and 19.01 ± 4.36, respectively. The detailed sub-dimensional statistical results for each indicator are shown in Table 3. In terms of scores, 284 (26.4%) of RTMSs had low willingness to fulfill the contract, 337 (31.3%) had medium willingness, and 455 (42.3%) had high willingness. “In the future, I will leave the medical industry during the term of my contract” (5.21 ± 1.08) and “I often want to give up studying medicine specialty” (4.82 ± 1.33) were higher than other items.

**Table 3:** Item scores in honesty-credit, specialty identity, career identity, and willingness to fulfill the contract.

**Items**	**Mean ±SD**
**Honesty-credit**	94.04 ± 10.25
Self-honesty-credit consciousness	25.64 ± 4.17
Evaluation of the honesty-credit of college students	25.68 ± 3.75
Influencing factors of college students' honesty-credit	19.79 ± 3.01
Measures to improve the honesty-credit of college students	22.94 ± 4.09
**Specialty identity**	**88.35** **±14.26**
Cognitive	19.40 ± 3.63
Emotional	31.63 ± 6.13
Behavioral	23.07 ± 4.08
Appropriate	14.25 ± 3.12
**Career identity**	**57.49** **±9.79**
Sense of professional benefit	23.21 ± 4.60
External influencing factors for professional	15.82 ± 2.89
Sense of professional motivation	18.46 ± 3.59
**Willingness to fulfill the contract**	**19.01** **±4.36**
I want to leave the specialty of rural-oriented tuition-waived medicine and switch to other medical specialties.	4.25 ± 1.56
I often want to give up studying medicine specialty	4.82 ± 1.33
In future, I will leave the orientated rural hospital during the term of my contract.	4.73 ± 1.39
In future, I will leave the medical industry during the term of my contract.	5.21 ± 1.08

### Correlations of study variables

Table 4 presents Pearson's correlation coefficients of the four main observed variables of RTMSs. Honesty-credit was significantly positively correlated with willingness to fulfill the contract, specialty identity, and career identity. Specialty identity was also positively correlated with career identity.

**Table 4:** Correlation coefficients among study variables.

**Items**	**Honesty-credit**	**Specialty identity**	**Career identity**
Honesty-credit			
Specialty identity	0.514^**^		
Career identity	0.555^**^	0.792^**^	
Willingness to fulfill the contract	0.365^**^	0.429^**^	0.483^**^

### Testing of the constructed study model

**Figure 2:**
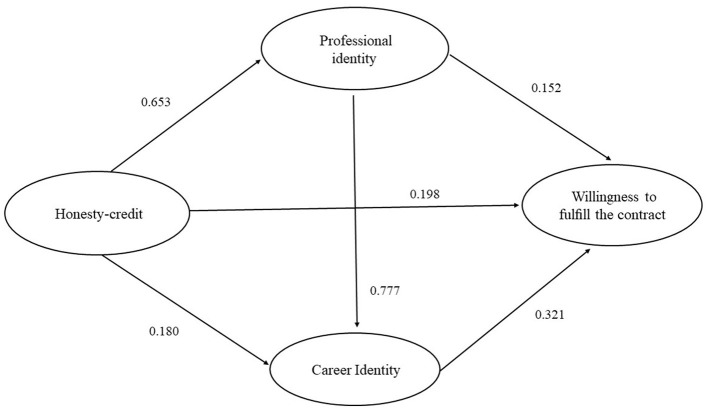
Final model and standardized model paths.

An SEM was established to connect, measure, and evaluate the links among four variables (honesty-credit, specialty identity, career identity, and willingness to fulfill the contract). The generalized least square method was used to fit the data into the previously constructed theoretical model, and the model was modified and improved according to the final fitting index. Finally, the constructed model showed the relationship between the path validity of the four variables with each other (Fig. 2). The fitting indexes of the final modified hypothesis model were GFI = 0.937, AGFI = 0.926, NFI = 0.947, IFI = 0.955, CFI = 0.936, TLI = 0.945, and RMSEA = 0.063. All the indexes met the requirement of the reference value, indicating that the model fitted well.

Using maximum likelihood estimation, each path was corrected by 2000 deviations and repeatedly guided. Figure 2 and Table 5 presents the mediation analysis path and effect values. Honesty-credit had a significant direct positive effect on willingness to fulfill the contract (β = 0.198, *P* < 0.001), specialty identity (β = 0.653, *P* < 0.001), and career identity (β = 0.180, *P* < 0.001). Specialty identity had a direct positive effect on career identity (β = 0.777, *P* < 0.001), and career identity also had a direct positive effect on willingness to fulfill the contract (β = 0.321, *P* < 0.001).

**Table 5:** Significance test of the mediating test.

**Model pathways**	**Estimated**	**95%CI**
**Total effects**		
Specialty identity ← Honesty-credit	0.653	0.560–0.736
Career identity ← Honesty-credit	0.688	0.615–0.755
Willingness to fulfill the contract ← Honesty-credit	0.518	0.427–0.601
Willingness to fulfill the contract ← Specialty identity	0.401	0.255–0.538
Willingness to fulfill the contract ← Career identity	0.321	0.028–0.626
Career identity ← Specialty identity	0.777	0.631–0.918
**Direct effects**		
Specialty identity ← Honesty-credit	0.653	0.560–0.736
Career identity ← Honesty-credit	0.180	0.028–0.338
Willingness to fulfill the contract ← Honesty-credit	0.198	0.040–0.353
Willingness to fulfill the contract ← Specialty identity	0.152	−0.182 to 0.441
Willingness to fulfill the contract ← Career identity	0.321	0.028–0.626
Career identity ← Specialty identity	0.777	0.631–0.918
**Indirect effects**		
Career identity ← Honesty-credit	0.507	0.373–0.652
Willingness to fulfill the contract ← Honesty-credit	0.320	0.219–0.428
Willingness to fulfill the contract ← Specialty identity	0.250	0.034–0.556

Table 6 shows the important test quantification results of three mediated pathways between the four factors. In the intermediary path between honesty-credit and willingness to fulfill the contract, career identity had significant mediating effects (95% CI: 0.007–0.051). Career identity also had significant mediating effects between specialty identity and willingness to fulfill the contract (95% CI: 0.030–0.149), and specialty identity had significant mediating effects between honesty-credit and career identity (95% CI: 0.465–0.760).

**Table 6:** Significance test of every mediating pathway.

**Model pathways**	**95% CI**
Career identity ← Specialty identity ← Honesty-credit	0.465–0.760
Willingness to fulfill the contract ← Career identity ← Honesty-credit	0.007–0.051
Willingness to fulfill the contract ← Career identity ← Specialty identity	0.030–0.149

## Discussion

A series of problems existing in China's rural medical system, such as insufficient physician rural practice, weak crisis awareness, and insufficient capacity of rural doctors, have been exposed in various stages of COVID-19 prevention and control (28, 29). The original goals of the RTME project included training a group of rural grassroots general practitioners capable of providing clinical treatment, public health crisis emergency response, and infectious disease prevention and treatment. These objectives were set with the aim of realizing the goal of “ensuring basic, strengthening grassroots, and building mechanism” in rural areas of China. However, our study found that only 455 (42.3%) RTMSs had a high willingness to fulfill the contract. At the same time, it was confirmed that honesty-credit, professional identity, and career identity were early and powerful predictors of the willingness to fulfill the contract of RTMSs.

The employment direction and employment positions were defined through an agreement signed prior to RTMS enrollment. The extent to which the project can achieve its intended outcomes is greatly influenced by the students' willingness to fulfill the contract. Due to the pressure of penalties for breach of contract, such as financial penalties and the recording of personal breach of trust records, the contract fulfillment data of RTMS graduates in rural grassroots employment after graduation were relatively ideal. The actual employment compliance rate of RTMSs was more than 80% in both developed areas in Eastern China and underdeveloped areas in Western China (12, 17, 30, 31). However, compared with the actual employment rate, the willingness to fulfill the contract rate of RTMSs studying at school to get employed in rural areas was extremely low. It was only 33.58% in some cases (31). This indicated that the employment of RTMS at the grassroots level was not voluntary but more due to the pressure of breach of contract. This situation also posed significant risks to their intention to quit after the contract expires and the overall success or failure of RTME implementation (12). It was seen that, how to correctly carry out the education of specialty identity and career identity, strengthen the willingness to fulfill the contract, and make RTMSs realize the contract fulfillment based on honesty and voluntariness was the key to the sustainable development of the RTME and themselves. This approach was crucial to truly improve the health of rural residents, which was not only necessary but also aligned with the original intention of our research (32).

The structural equation proved that the honesty-credit of RTMSs had a direct positive effect on willingness to fulfill the contract and an indirect positive effect through the mediating effect of career identity. In modern society, honesty-credit refers to the contract that should be abided by to maintain the interests of both parties when they are constrained by various interests and situations in the process of interpersonal communication (28). Therefore, honesty-credit goes through the process of “judgment → commitment and commitment → practice” (28). Honesty-credit is the basis of college students' employment. With the growing challenges in college student employment, many scholars have begun to pay attention to the honesty-credit of college students. It is believed that the lack of honesty is one of the factors contributing to the difficulties faced by college students in securing employment. Dishonest behaviors such as cheating and breaching contracts have had profoundly negative impacts on the students themselves, their educational institutions, and potential employers. These behaviors significantly impede the overall employment prospects of college students (29). Medical students bear the special work of curing diseases and saving lives, and their honesty directly affects patients' trust in doctors and doctor–patient harmony. However, as a special group of medical students, the honesty-credit of RTMSs was currently receiving insufficient attention from researchers. First of all, many RTMSs lacked an understanding of the national RTME policy, and their specialty identity had not been successfully established, directly affecting their enthusiasm for learning. Second, some students were too optimistic. They thought that they had signed a contract with the orientation unit and got a job, leading to a lack of learning motivation and burnout. However, after graduation, their post-ability was obviously insufficient to undertake rural primary medical and health services, which was also a manifestation of dishonesty (33–35). Furthermore, due to the disparities between the rural medical environment and urban hospitals, some RTMSs exhibited a limited sense of specialty identity toward publicly funded general practice. Consequently, their level of career identity as rural doctors was also notably low. They chose to directly break the contract and refuse to serve the grassroots level, which constituted another breach of honesty-credit. Therefore, these two cases were dishonest behavior, which not only affected the construction of the team of general practitioners in rural areas but also led to the failure of the RTME project and the loss and wastage of the national investment.

Specialty identity refers to learners' emotional acceptance and recognition of their specialty based on their understanding of their own major, accompanied by positive external behavior and an internal sense of appropriateness. Students with higher specialty identities had greater recognition and acceptance of their major. They were also more inclined to view their specialty as an integral part of their future career and were willing to exert efforts toward its success (36, 37). Western scholars equated specialty identity with career identity and found that specialty identity was also significantly correlated with individual mental health level and turnover intention (37–40). Domestic studies also found that the degree of university students' recognition of their specialty affected their learning enthusiasm, investment level, professional ability, and future career direction (41, 42). According to the structural equation of this study, specialty identity had no direct influence on their performance intention. However, it had a positive influence through the mediating effect of career identity. The cultivation of knowledge structure, ability level, and quality of RTMSs was closely related to the implementation effect of the national primary medical and health policy (7). The career identity of RTMSs not only affected their academic performance and future career behavior but also further affected the implementation effect of the national policy on strengthening primary medical and health talents. By investigating 275 RTMSs, Zhang Tingjian et al. found that the total average score of career identity and the score of four dimensions of directed medical students were lower than those of ordinary medical students (38), which was extremely similar to our results. The average score of career identity of RTMSs calculated by us was 3.841 ± 0.385. The scores of each dimension from high to low were as follows: emotional (3.954 ± 0.587), cognitive (3.880 ± 0.527), behavioral (3.845 ± 0.463), and appropriateness (3.563 ± 0.608). Except for the emotional score, the scores in the other dimensions were lower compared with those of ordinary medical students. This may be related to the fact that RTMSs chose the RTME specialty primarily due to guaranteed employment opportunities, tuition waivers, and relatively lower admission scores rather than pursuing it out of genuine interest and passion. However, RTMSs gradually realized the limitations of this specialty in employment and postgraduate entrance examination compared with students of other specialties as the grade increased. Consequently, their scores of specialty identity decreased. In addition, some RTMSs, especially many rural students, were gradually unwilling to return to rural primary medical institutions to fulfill their commitments after adapting to life in big cities. Meanwhile, schools did not further keep up with the professional ideological education of students, leading to a decline in the professional recognition of RTMSs (43–47).

As a psychological concept, career identity refers to an individual's positive evaluation of the goals, attractiveness, and social value of a current or future career (48–50). RTMSs' career identification with future rural doctors directly determines their enthusiasm to study in school, intention to serve in primary medical institutions, and future work attitude (49). The SEM results showed that RTMSs' career identity had a direct positive impact on willingness to fulfill the contract. Additionally, it was found to have a positive indirect impact between honesty-credit and willingness to fulfill the contract, as well as between specialty identity and willingness to fulfill the contract. However, the career identity of RTMSs was worse than that of publicly funded normal university students (51) and other no-publicly funded medical students (52). One reason was that, compared with urban doctors, rural doctors had a cumbersome professional situation. They were not only responsible for diagnosing and treating rural residents, but also responsible for establishing health records, chronic disease management, health education, planned immunization, and other basic public health services. Compared with the staff of city-county medical institutions with the same qualifications, rural doctors had poor working and living conditions, restricted movement of staff, delayed salary subsidies, and bottlenecks in career development, which affected the professional identity of RTMSs. Moreover, with the development of economic levels and the convenience of urban and rural transportation, many residents chose to go to large hospitals for medical treatment and gradually lost their trust in rural doctors. A large number of rural general practitioners had changed jobs and resigned, resulting in a large number of vacancies (25).

To sum up, this study revealed three influencing paths of the willingness to fulfill the contract of RTMSs using the structural equation. As a warning factor, honesty-credit, specialty identity, and career identity could accurately predict the willingness to fulfill the contract of RTMSs. The results further verified the complex influence of honesty-credit on the willingness to fulfill the contract of RTMSs. First, honesty-credit had a direct positive effect on willingness to fulfill the contract. Second, honesty-credit could also indirectly affect willingness to fulfill the contract through the intermediary effect of career identity. Third, honesty-credit affected career identity through specialty identity and then affected the willingness to fulfill the contract. This suggested that a more intricate mechanism existed between honesty-credit and willingness to fulfill the contract. However, the academic research on the willingness to fulfill the contract of RTMSs is insufficient. In future, we should not only pay attention to the factors affecting the contract fulfillment of RTMSs but also construct perfect intervention measures for the breach of contract by RTMSs from the perspective of the predictive factors to improve the contract of fulfillment rate. In addition, as a subjective and complex multidimensional variable, a detailed analysis of the willingness to fulfill the contract of RTMSs through quantitative research is difficult. In future, qualitative research methods should be used to discuss the contract fulfillment of medical students at the micro and deep levels (53–58).

## Conclusion

The training of RTMSs had not only the commonness of medical education but also its unique personality. Specialty cognition and identity of RTMSs affected their career identity, which in turn affected their willingness to fulfill the contract, learning motivation, and work enthusiasm. It was suggested that the publicity of the RTME program needs to be comprehensively strengthened by universities and admissions departments during the admissions process, and the teachers and parents should make students choose their majors and careers voluntarily according to their personalities, interests, and characteristics, which is conducive to the sustainable development of RTME project. In the process of training, rural general practitioners' career identity education should be vigorously carried out in schools, and students should be encouraged to participate in the survey of rural primary medical institutions, so as to enable RTMSs to understand the nature and scope of rural general practitioners' work and better define the career direction of rural general practitioners.

Compared with other medical students, the emphasis on honesty-credit education for RTMSs is of greater significance. This is because, upon graduation, they are required to serve in rural medical institutions where conditions and benefits are relatively limited. Through integrity education, RTMSs can gain a clear understanding of the essential contribution and significance of their role to the country, society, and rural communities. This enables them to establish a stronger sense of professional identity and actively fulfill their responsibilities as rural doctors.

The support and guarantee for RTMSs need to be strengthened by the state and the government. The protection and treatment of grassroots medical and health workers need to be improved, and reasonable mobility, appointment, promotion, and training mechanisms need to be established, so as to encourage excellent grassroots medical and health workers to stay in rural communities.

## Data availability statement

The original contributions presented in the study are included in the article/Supplementary material, further inquiries can be directed to the corresponding author.

## Ethics statement

The studies involving human participants were reviewed and approved by the Jining Medical University Institutional Review Board (JNMC-2022-YX-030). Written informed consent from the participants was not required to participate in this study in accordance with the national legislation and the institutional requirements.

## Author contributions

XZ and BS designed this study. ZT, XC, CZ, BY, CW, and QL collected the data and conducted the analysis. XZ and YZ wrote the first version of the manuscript. All authors interpreted the findings and critically evaluated and edited the manuscript. All authors approved the final draft for publication.
